# The use of corticosteroids in patients with COPD or asthma does not decrease lung squamous cell carcinoma

**DOI:** 10.1186/s12890-015-0153-5

**Published:** 2015-12-03

**Authors:** Zhi-Hong Jian, Jing-Yang Huang, Frank Cheau-Feng Lin, Oswald Ndi Nfor, Kai-Ming Jhang, Wen-Yuan Ku, Chien-Chang Ho, Chia-Chi Lung, Hui-Hsien Pan, Yu-Chiu Liang, Ming-Fang Wu, Yung-Po Liaw

**Affiliations:** 1 Department of Public Health and Institute of Public Health, Chung Shan Medical University, Taichung, Taiwan; 2 School of Medicine, Chung Shan Medical University, Taichung, Taiwan; 3 Department of Thoracic Surgery, Chung Shan Medical University Hospital, Taichung, Taiwan; 4 Department of Neurology, Changhua Christian Hospital, Changhua, Taiwan; 5 Department of Physical Education, Fu Jen Catholic University, New Taipei City, Taiwan; 6 Department of Family and Community Medicine, Chung Shan Medical University Hospital, No. 110, Sec. 1 Jianguo N. Rd., Taichung City, 40201 Taiwan; 7 Department of Pediatrics, Chung Shan Medical University Hospital, Taichung, Taiwan; 8 College of Humanities and Social Sciences, Taipei Medical University, Taipei City, Taiwan; 9 Divisions of Medical Oncology and Pulmonary Medicine, Chung Shan Medical University Hospital, Taichung, Taiwan

**Keywords:** Asthma, Chronic obstructive pulmonary disease, Corticosteroids, Lung squamous cell carcinoma

## Abstract

**Background:**

Asthma and COPD (chronic obstructive pulmonary disease) lead to persistent airway inflammation and are associated with lung cancer. The objective of the study was to assess the relationship between inhaled (ICS) and oral corticosteroid (OCS) use, and risk of lung squamous cell carcinoma (SqCC).

**Methods:**

This study was a nested case–control study. Patients with newly diagnosed asthma or COPD between 2003 and 2010 were identified from the National Health Insurance Database. Cases were defined as patients diagnosed with SqCC after enrollment. For each case, four control individuals who were randomly matched for sex and age and date diagnosis of asthma or COPD were selected.

**Results:**

From the 1,672,455 eligible participants, 793 patients with SqCC were matched with 3,172 controls. The odds ratios (ORs) of SqCC in men who received high and low-dose ICS were 2.18 (95 %CI, 1.56–3.04) and 1.77 (1.22–2.57), respectively. Similarly, the ORs were 1.46 (95 %CI, 1.16–1.84) and 1.55 (95 %CI, 1.22–1.98) for men who were placed on low and high dose OCS. However, there was no significant association between cumulative ICS and/or OCS and risk of SqCC in women. Recent dose increase in corticosteriod was significantly associated with risk of SqCC. Specifically, among men, the ORs for SqCC were 8.08 (95 %CI, 3.22–20.30) for high-dose ICS + OCS, 4.49 (95 % CI, 2.05–9.85) for high-dose ICS, and 3.54 (95 % CI, 2.50–5.01) for high-dose OCS treatments, respectively. The OR for SqCC in women who received high-dose OCS was 6.72 (95 %CI, 2.69–16.81).

**Conclusion:**

Corticosteroid use did not decrease SqCC in patients with asthma or COPD. Recent dose increase in corticosteroids was associated with SqCC.

## Background

Squamous cell carcinoma (SqCC) accounts for approximately 20 % of all lung cancers in the United States [1]. It is the predominant histological type of lung cancer in men [2]. Lung cancer has been linked with life expectancy losses in Taiwan [3]. Chronic inflammation and frequent pulmonary exacerbations may result in repeated injury and repair which can lead to a high cell turnover, DNA damage, malignant cell transformation, and ultimately, development of lung cancer [4]. Asthma and chronic obstructive pulmonary disease (COPD) are chronic airway inflammatory diseases commonly associated with lung cancer [5, 6]. Severe airflow obstruction is an independent risk factor for lung cancer [7, 8]. The prevalence of asthma (11.9 %) and COPD (2.48 %) in Taiwan is high [9, 10].

Oral (OCS) and inhaled corticosteroid (ICS) have reduced local and systemic inflammation among patients with asthma or COPD [11, 12]. However, studies to assess histologic type of lung cancer among ICS and OCS users are limited. In this study, we investigated the association between corticosteroids and SqCC.

## Methods

### Data source

Data used in this study were obtained from 2003 to 2010 using the National Health Insurance Research Database (NHIRD). Taiwan’s National Health Insurance covers more than 99 % of the 23 million residents and contains enrollment files, claims data, catastrophic illness files, and registry for treatments. The database is one of the largest datasets described in most epidemiological studies [13–15]. This study used multiple databases: the NHIRD, Taiwan Cancer Registry Database (TCRD), and National Death Registry Database (NDRD) with permission of the Department of Statistics, Ministry of Health and Welfare of Taiwan. The source data was encrypted and the data extracted was anonymous. This study was approved by the Institutional Review Board of the Chung-Shan Medical University Hospital, Taiwan.

### Study design

A nested case–control study was conducted to overcome the time-varying nature of corticosteroid treatment. Analytic data included subjects aged ≥20 years with newly diagnosed asthma or COPD from 2003 to 2010. The date of the first diagnosis of asthma or COPD was defined as the initiation date. Excluded were individuals with incomplete information on sex and registry data. Also excluded were individuals diagnosed with lung cancer before 2002, initiation date, or 2 years after the initiation date.

### Identification of patients with lung cancer

The study began in 2003. Cases with newly diagnosed lung cancer were followed until death, loss to follow-up, or the study end in 2010. Lung cancer was identified using the International Classification of Diseases, Ninth Revision, Clinical Modification (ICD-9-CM) code 162. The index date was defined as the date of first assignment of the above ICD codes.

### Case definition

The TCRD was used to confirm cell types of lung cancer. All major cancer care hospitals in Taiwan are obligated to submit cancer type, initial tumor stages and histology to Taiwan Cancer Registry funded by the Ministry of Health and Welfare [16]. Lung cancer was coded by ICD-9-CM 162 or ICD 10 C34.0, C34.1, C34.2, C34.3, C34.8, and C34.9. Morphological diagnoses were made using the ninth revision of the International Classification of Diseases for Oncology, based on codes 80522, 80523, 80702, 80703, 80713, 80723, 80733, 80743, 80763, 80823, 80833, and 80843 for SqCC.

### Control definition

For each case, up to four control individuals, who were randomly matched for sex, age diagnosed with asthma or COPD, and initiation date were selected without replication from individuals without lung cancer.

The NDRD, NHIRD, and TCRD were used to assess the age at cancer onset, person-year follow-up, death and survival time, and potentially unconfirmed cases diagnosed with cancer.

### Inhaled and oral corticosteroid exposure

We identified patients who were prescribed OCS and ICS between the initiation and index date. Data were collected on prescription dates, prescribed daily dose, and the number of days supplied. In accordance with the Anatomic Therapeutic Chemical classification of ICS drugs, beclomethasone, budesonide, fluticasone, and ciclesonide were selected as the major drugs of interest, whether dispensed alone or in combination with an inhaled β2 agonist. The defined daily dose (DDD) recommended by the World Health Organization is a unit for measuring a prescribed amount of drug to standardize the comparison of drug usage between different drugs [17]. All ICS were compared using the following equation: total amount of a drug/DDD of a drug = number of DDDs [18]. Cumulative DDD (cDDD), which encompasses both dosage and duration of exposure, was estimated as the sum of the dispensed DDDs of any ICS and was used to correlate ICS use with SqCC risk.

All OCS prescriptions were converted to hydrocortisone equivalents (4 mg of hydrocortisone = 1 mg of prednisolone = 5 mg of cortisone = 0.8 mg methylprednisolone = 0.8 mg of triamcinolone = 0.4 mg of paramethasone = 0.15 mg of betamethasone = 0.15 mg of dexamethasone) [19].

An average quarter dose was calculated by dividing the total number of milligrams of OCS or cDDDs of ICS by the number of quarter prescribed during the assessment period.

### Covariates

The diagnoses of pulmonary diseases and comorbidities were confirmed by either 2 outpatient visits or one hospitalization in one year. Baseline pulmonary diseases and other comorbidities are listed as follows: asthma (ICD-9-CM: 493), COPD (ICD-9-CM: 490, 491, 492, 494, and 496), pulmonary tuberculosis (ICD-9-CM: 010–012, and 137.0), pneumonia (ICD-9-CM: 486), chronic renal disease (ICD-9-CM: 585 and 586), hyperlipidemia (ICD-9-CM: 272), and smoking-related cancers (ICD-9-CM: 140–150, 157, 160–161, and 189). Type 2 DM (ICD-9-CM: 250, excluding type I DM) is characterized by hyperinsulinemia in the context of insulin resistance and an increased level of circulating insulin-like growth factor 1 which are associated with an increased risk of cancer [20]. Individuals with Type 1 DM were excluded because of exposure to lower levels of exogenously administered insulin. As a proxy of COPD or asthma severity, we assessed the number of outpatient and inpatient visits between the initiation and index date. However, information regarding lifestyle or behavior such as smoking was not recorded in the NHIRD, hence preventing direct adjustment of possible confounders.

### Statistical analysis

Data analyses were made using SAS 9.3 software (SAS Institute, Cary, NC). Chi-square and t tests were used to compare baseline sociodemographic characteristics and comorbidities between cases and controls. Conditional logistic regression was used to assess the association between corticosteroid use and SqCC. Low or high-dose corticosteroid was defined by the median of corticosteroid dose per quarter. Adjusted odd ratios (ORs) were presented with 95 % confidence intervals (CIs) and a *P* value of less than 0.05 was considered statistical significance.

## Results

We identified 2,384,046 individuals with first-time diagnosis of asthma or COPD from 2003 to 2010. The total number of individuals excluded were as follows; <20 years of age (*n* = 559,589), incomplete data on sex (*n* = 32,914) and registration (*n* = 104,499), type 1 DM (*n* = 123), diagnosed with lung cancer before 2002 or initiation date, and 2 years after the initiation date (*n* = 14,466). The final enrolment included 1,672,455 individuals. A total of 4032 patients were identified with lung cancer, 793 of whom were patients with SqCC.

### Patients with SqCC and controls

The baseline characteristics of patients with SqCC (793) and their controls (3172) are summarized in Table 1. The sample size comprised 87.4 % of men. Cases had comparatively low income, health care utilities, pneumonia, pulmonary tuberculosis, smoking-related cancers, and ICS and OCS use.

**Table 1:** Baseline characteristics of controls and cases with lung squamous cell carcinoma

	Control (*N* = 3172)	Case (*N* = 793)	*P*-value
Sex (%)			1.000
Men	2772 (87.4)	693 (87.4)	
Women	400 (12.6)	100 (12.6)	
Low income (%)			0.028
No	3133 (98.8)	775 (97.7)	
Yes	39 (1.2)	18 (2.3)	
Urbanization (%)			0.012
Urban	1593 (50.2)	356 (44.9)	
Suburban	1082 (34.1)	286 (36.1)	
Rural	497 (15.7)	151 (19.0)	
Age diagnosed with asthma or COPD (year) (mean ± sd)	71.6 ± 9.4	71.6 ± 9.4	1.000
Months between initiation and index date (mean ± sd)^a^	46.3 ± 16.3	46.3 ± 16.3	1.000
No. of health care utilities between initiation and index date			
No. of outpatient visits for asthma (%)			
0–10	2925 (92.2)	698 (88.0)	<0.001
>10	247 (7.8)	95 (12.0)	
No. of hospitalization for asthma (%)			
0–2	3128 (98.6)	777 (98.0)	0.193
>2	44 (1.4)	16 (2.0)	
No. of outpatient visits for COPD (%)			
0–10	2800 (88.3)	622 (78.4)	<0.0001
>10	372 (11.7)	171 (21.6)	
No. of hospitalization for COPD (%)			
0–2	2995 (94.4)	683 (86.1)	<0.0001
>2	177 (5.6)	110(13.9)	
Comorbidities (%)			
Pneumonia	1179 (37.2)	483 (60.9)	<0.0001
Pulmonary tuberculosis	214 (6.7)	132(16.7)	<0.0001
Chronic renal disease	302 (9.5)	90 (11.4)	0.123
Diabetes mellitus	1035 (32.6)	271 (34.2)	0.408
Hyperlipidemia	999 (31.5)	213 (26.9)	0.011
Smoking-related cancers	63 (2.0)	40 (5.0)	<0.0001
Medication within 2-year prior to index date^b^			
ICS, cDDDs per quarter			<0.0001
No use	2866 (90.4)	607 (76.5)	
Lower dose (≦18.8)	156 (4.9)	95 (12.0)	
Higher dose (>18.8)	150 (4.7)	91 (11.5)	
OCS (Hydrocortisone equivalent/quarter)			<0.0001
No use	1955 (61.6)	338 (42.6)	
Lower dose (≦90.0 mg)	644 (20.3)	195 (24.6)	
Higher dose (>90.0 mg)	573 (18.1)	260 (32.8)	
Aspirin (mg per quarter)			0.1888
No use	1998 (63.0)	489 (61.7)	
Lower dose (≦3012.5)	574 (18.1)	165 (20.8)	
Higher dose (>3012.5)	600 (18.9)	139 (17.5)	

*cDDD* cumulative defined daily dose, *COPD* chronic obstructive pulmonary diseases, *ICS* inhaled corticosteroid, *OCS* oral corticosteroid, *sd* standard deviation

^a^Initiation date was defined as the date asthma or COPD was diagnosed while index date was defined as the date lung cancer when diagnosed

^b^Low and high dose medications were defined by the median dose of medications

### ICS and OCS and the risk of developing SqCC

Compared with non-ICS users (Model 1), the ORs for SqCC in low and high-dose ICS were 2.09 (95 % CI, 1.52–2.88) and 1.88 (95 % CI, 1.32–2.66), respectively (Table 2). Similarly, the ORs for SqCC in low and high-dose OCS users were 1.48 (95 % CI, 1.20–1.83) and 1.54 (95 % CI, 1.22–1.93), respectively. Specifically, among men (Model 2), the ORs for SqCC were 2.18 (95 % CI, 1.56–3.04) and 1.77 (1.22–2.57) in low and high-dose ICS, and 1.46 (95 % CI, 1.16–1.84) and 1.55 (95 % CI, 1.22–1.98) in low and high-dose OCS users, respectively. However, there was no increased risk of SqCC among women who received ICS and OCS.

**Table 2:** Risk of developing squamous cell carcinoma based on the cumulative dose of ICS and OCS

	Mode 1		Model 2			
	All		Male		Female	
	OR (95 % CI)	*p*-value	OR (95 % CI)	*p*-value	OR (95 % CI)	*p*-value
Medication within 2-year prior to index date^a^						
ICS (cDDDs per quartier)						
No use	1	-	1	-	1	-
Lower dose (≦18.8)^b^	**2.09** (1.52–2.88)	<.0001	**2.18** (1.56–3.04)	<.0001	1.16 (0.35–3.85)	0.812
Higher dose (>18.8)	**1.88** (1.32–2.66)	<0.001	**1.77** (1.22–2.57)	0.003	2.96 (0.87–10.04)	0.082
OCS (Hydrocortisone equivalent/quarter)						
No use	1	-	1	-	1	-
Lower dose (≦90.0 mg)^b^	**1.48** (1.20–1.83)	<0.001	**1.46** (1.16–1.84)	0.001	1.59 (0.88–2.87)	0.124
Higher dose (>90.0 mg)	**1.54** (1.22–1.93)	<0.001	**1.55** (1.22–1.98)	<0.001	1.51 (0.75–3.04)	0.253

Each model was adjusted by low income, urbanization, health care utility, comorbidities and aspirin use

*cDDD* cumulative defined daily dose, *CI* confidence interval, *ICS* inhaled corticosteroid, *OCS* oral corticosteroid, *OR* odds ratio

^a^Index date was defined as the date of lung cancer diagnosis

^b^Low and high-dose ICS and OCS were defined by the median of cumulative ICS and OCS dose (18.8 DDD / quarter and 90 mg hydrocortisone/quarter, respectively).

Significant data are presented in bold font

### Risk of SqCC in patients with increased dose of corticosteroids

In Table 3, recent dose increase in corticosteriods within 3 months prior to index date was significantly associated with SqCC (Model 3). The adjusted ORs of SqCC were 8.01 (95 % CI, 3.38–19.01) in high-dose ICS + OCS, 4.14 (95 % CI, 1.98–8.66) in high-dose ICS, and 3.77 (95 % CI, 2.75–5.16) in high-dose OCS users. Specifically, among men (Model 4), the ORs for SqCC were 8.08 (95 % CI, 3.22–20.30) in high-dose ICS + OCS, 4.49 (95 % CI, 2.05–9.85) in high-dose ICS, and 3.54 (95 % CI, 2.50–5.01) in high-dose OCS users. Among women (Model 4), the OR for SqCC was 6.72 (95 % CI, 2.69–16.81) in high-dose OCS users. There was no significant interactions between ICS, OCS and SqCC (*P* = 0.234). The interactions between sex and corticosteroids were also not significant (*P* = 0.764).

**Table 3:** Adjusted risk for squamous cell carcinoma in patients with recent dose-increase in corticosteroids

Months before index date^a^		Model 3			Model 4	
−6 - -3	−3 – 0	All case			Men	Women
		Control	Case	OR (95 % CI)	OR (95 % CI)	OR (95 % CI)
SqCC						
ICS_L_ + OCS_L_	ICS_L_ + OCS_L_	2589	475	1	1	1
ICS_L_ + OCS_L_	ICS_L_ + OCS_H_	145	107	**3.77** (2.75–5.16)	**3.54** (2.50–5.01)	**6.72** (2.69–16.81)
ICS_L_ + OCS_L_	ICS_H_ + OCS_L_	20	18	**4.14** (1.98–8.66)	**4.49** (2.05–9.85)	3.65 (0.26–51.94)
ICS_L_ + OCS_L_	ICS_H_ + OCS_H_	14	22	**8.01** (3.38–19.01)	**8.08** (3.22–20.30)	3.77 (0.24–60.39)
P for ICS × OCS interaction = 0.234						

Each model was adjusted for low income, urbanization, health care utility, comorbidities, and aspirin use

Low and high-dose ICS and OCS were defined by the median of cumulative ICS and OCS dose (18.8 DDD/quarter and 90 mg hydrocortisone/quarter, respectively)

*CI* confidence interval, *ICS* inhaled corticosteroid, *ICS*_*H*_ high cumulative dose of inhaled corticosteroid, *ICS*_*L*_ low cumulative dose of inhaled corticosteroid, *OCS* oral corticosteroid, *OCS*_*H*_ high cumulative dose of oral corticosteroid, *OCS*_*L*_ low cumulative dose of oral corticosteroid, *OR* odds ratio

^a^Index date was defined as the date of lung cancer diagnosis

Significant data are presented in bold font

## Discussion

Over the past decade, some studies have documented a possible link between chronic inflammatory lung diseases and lung cancer [5, 6]. Corticosteroids are used to control persistent airway inflammation in patients with COPD or asthma. However, the impact of corticosteroids on the specific types of lung cancer has not been addressed. In this study, cumulative doses of OCS and ICS were associated with SqCC in men. Our observation also showed that SqCC was associated with a substantial increase in steroid use in the preceding 3 months. However, it is far too short a time frame to be biologically plausible that steroids are causing the cancer. High-dose steroids are the standard treatment in acute exacerbation of respiratory symptoms that may serve as risk factors for SqCC.

Our results demonstrated that patients with SqCC had significantly higher pneumonia, pulmonary tuberculosis, and smoking-related cancers, but lower hyperlipidemia. Because national databases do not contain detailed information regarding smoking history, occupational exposures, and other risk factors of lung cancer, other diseases (excluding COPD and asthma) may affect the risk of lung SqCC. In a cohort study with 17,859,318 Taiwanese residents, Jian et al. evaluated gender disparities in pulmonary diseases, comorbidities, and effects on SqCC [5]. A total of 6,637 cases of SqCC (male/female: 5877/760) were identified. Among men, TB and smoking-related cancers were associated with increased risk of SqCC. However, hyperlipidemia was associated with a decreased risk. Among women, TB, low income, type 2 DM, and smoking-related cancers were attributed to increased risk of SqCC. In another study involving 22,034 patients with pneumococcal pneumonia, the hazard ratio (HR) of lung cancer was 4.24 (95 % CI, 3.96–4.55) [21]. These results are consistent with our investigations.

With effective treatment and control of allergens and irritants, majority of patients with asthma or COPD have a controlled disease though some patients can still be exposed to frequent exacerbations [22, 23]. ICS therapy forms the basis for treatment of asthma and COPD, improving disease control and reducing exacerbations [24–26]. Acute severe exacerbations require the addition of OCS to control increased inflammation and respiratory symptoms [22]. Although corticosteroids are powerful nonspecific anti-inflammatory agents, they have little effect on biopsy proven inflammation and did not change the rate of decline of pulmonary function [23]. Airways hyper-responsiveness, remodeling, and inflammation have persisted [27]. This indicates that corticosteroids can’t prevent airway inflammation that may lead to lung carcinogenesis.

Few studies on the relationship between corticosteroids and lung cancer have yielded conflicting results. Gundisch et al. found that glucocorticoids promoted tumor cell proliferation in a pre-clinical mouse model of lung carcinoma [28]. Budesonide produced 70 % inhibition of lung tumor multiplicity and 94 % reduction of total tumor in A/J mice [29]. Lee et al. analyzed new adult users of ICS (9177 cases and 37,048 controls) using the Korean national claims database [30]. Their study findings showed that ICS use had a significant linear association with a decreased lung cancer incidence. The adjusted OR was 0.79 (95 % CI, 0.69–0.90). In a study involving 10,474 United State veterans with COPD and a median follow-up of 3.8 years, a dose-dependent decreased risk of lung cancer was associated with ICS (triamcinolone >1,200 ug/day: adjusted HR, 0.39; 95 % CI, 0.16–0.96) [31]. However, after excluding participants who were diagnosed with lung cancer in the first year after enrollment, there was no significant reduction in lung cancer. In a nested case–control study involving patients (127 cases and 1470 controls) with newly diagnosed COPD who quitted smoking, and regular use of ICS and bronchodilators, the HRs for lung cancer were 0.50 (95 % CI, 0.27–0.90) in ICS + long acting beta agonist users and 0.64 (0.42–0.98) in ICS users compared with short-acting bronchodilators users [32].

However, none of these trials has evaluated the relationship between corticosteroids and specific types of lung cancer. Asthma and COPD have been closely associated with SqCC [5]. In this study, a recent dose increase in ICS and OCS is associated with SqCC. It is possible that lung cancer may be found after exacerbation of respiratory symptoms or treatment failure. An increased lung cancer risk was strongest 2 years after asthma was diagnosed [33]. Lung cancer is hard to diagnose. Prior to referral, a third of patients consulted their general practitioners three or more times with health problems related to lung cancer [34]. Diagnosis may be initially delayed because of symptomatic masks resulting from exacerbations of COPD and respiratory comorbidities [35, 36]. Prognosis of lung cancer is very poor. Longer diagnostic intervals have been associated with increased cancer stage and mortality [37]. In the presence of continuing or changing respiratory symptoms, doctors should be aware of the symptoms associated with lung cancer.

Our study results indicated no association between cumulative dose of corticosteroids and SqCC in women. In Taiwan, smoking is almost ten times more prevalent in men (45.7 %) than women (4.8 %) [38]. This might have influenced the observed differences in risk of developing SqCC between men and women. Except cigarette smoking, cooking fumes had been associated with female SqCC [39]. Sex hormones play a role in gender-based differences such as incidence, risk, histology, and pathogenesis of lung diseases, and may either contribute to pathogenesis of disease or serve as protective factors [40]. Besides, there were insufficient number of female patients to accurately analyze. More studies ought to be conducted to investigate the association between corticosteroids and SqCC among women.

This study has several strengths. First, the sample size is large with a long follow-up. It was based on nationwide databases, hence selection bias was possibly minimized. Second, to enhance the reliability of temporal relationship between corticosteroid use and SqCC, we excluded individuals who were diagnosed with lung cancer before 2002 and initiation date, or 2 years after initiation date. Nevertheless, this study had some limitations. First, corticosteroid exposure was assessed solely by refills recorded in the NHIRD, not by whether the subjects actually used their medication. Second, NHIRD, NDRD, and TCRD do not contain detailed information regarding smoking history, radon exposure, occupational exposures, diet preference, and family history, all of which may be risk factors for lung cancer.

## Conclusions

The use of corticosteroids in patients with asthma and COPD was associated with lung SqCC, especially in men. Recent dose-increase in corticosteroids was associated with SqCC. Lung cancer screening is necessary when treatment goals for asthma or COPD are not being met or when patients are not responding to current therapy.

## Abbreviation

cDDD: Cumulative defined daily dose
CI: Confidence interval
COPD: Chronic obstructive pulmonary disease
DDD: Defined daily dose
DM: Diabetes mellitus
HR: Hazard ratio
ICD-9-CM: International Classification of Diseases, Ninth Revision, Clinical Modification code
ICS: Inhaled corticosteroid
NDRD: National Death Registry Database
NHIRD: National Health Insurance Research Database
OCS: Oral corticosteroid
OR: Odds ratio
SqCC: Lung squamous cell carcinoma
TCRD: Taiwan Cancer Registry Database
